# Advanced oxidation protein products as prognostic biomarkers for recovery from acute kidney injury after coronary artery bypass grafting

**DOI:** 10.3109/1354750X.2012.690103

**Published:** 2012-03-09

**Authors:** Xinling Liang, Yuanhan Chen, Jian Zhuang, Min Zhang, Weiping Xiong, Huiming Guo, Fen Jiang, Penghua Hu, Dan Guo, Wei Shi

**Affiliations:** 1 Division of Nephrology, Guangdong General Hospital, Guangdong Academy of Medical Sciences, China; 2 Department of Cardiac Surgery, Guangdong Cardiovasclar Institute, Guangdong General Hospital, Guangdong Academy of Medical Sciences, China; 3 Department of Gastroenterology, the Sixth Afliated Hospital, Sun Yat-sen University, China, and; 4 Department of Pharmacology, Afliated Nanfang Hosipital, Nanfang Medical University, China

**Keywords:** Advanced oxidation protein products, acute kidney injury, prognostic biomarker, coronary artery bypass grafting, oxidative stress

## Abstract

Recovery from acute kidney injury (AKI) is related to long-term prognosis. This study, involving 56 patients with AKI and 56 controls from a prospective cohort undergoing coronary artery bypass grafting (CABG), investigated the prognostic performance of serum levels of advanced oxidation protein products (AOPPs) for predicting non-recovered AKI and non-completely recovered AKI. AOPP levels increased signifcantly 7 days after surgery in patients with non-recovered or non-completely recovered AKI. Increased AOPP levels were associated with both types of poor recovery from AKI. Results from receiver-operating characteristic (ROC) curves demonstrated that AOPP levels had good prognostic value for predicting non-recovered and non-completely recovered AKI.

## Introduction

Use of coronary artery bypass grafting (CABG) has been increasing rapidly over the last decade in China. Acute kidney injury (AKI) after CABG is common (Tuttle et al. 2003; Rosner & Okusa 2006). Epidemiologic evidences have suggested that not only the cardiac surgery-associated AKI (CSA-AKI) itself (Kangasniemi et al. 2008; Goldberg & Dennen 2008; Charytan et al. 2010), but also the lack of recovery from CSA-AKI are related to longterm adverse prognoses (Spurgeon-Pechman et al. 2007; Swaminathan et al. 2010; Srisawat et al. 2010). However, there are no efective treatments to improve renal recovery. One important barrier to progress in this area has been the lack of a reliable biomarker to predict recovery in individual patients. The ability to predict poor renal recovery would be extremely valuable for clinical decisions and to guide future research (Srisawat et al. 2011).

Prognostic biomarkers of AKI based on the physiology of renal recovery might also help to predict AKI recovery (Srisawat et al. 2010). However, few studies have stressed this issue, except a recent investigation using a panel of urine biomarkers (Srisawat et al. 2011). Cardiopulmonary bypass (CPB) during CABG is recognized as a cause of complex systemic infammatory and oxidative stress responses which signifcantly contribute to CSA-AKI and non-recovery of renal function (Gerritsen et al. 2001; Biglioli et al. 2003; Liu & Brakeman 2008; Staford-Smith et al. 2008; Srisawat et al. 2010). Advanced oxidation protein products (AOPPs) are oxidized proteins and are used widely as biomarkers of infammation and oxidative stress (Witko-Sarsat et al. 1998; Chen et al. 2011). AOPPs can activate infammatory cells (Witko-Sarsat et al. 1998) and contribute to the progression of renal failure (Li et al. 2007). Therefore, they are potential biomarkers for predicting renal recovery. In critically ill subjects, AOPP levels were shown to be higher in AKI patients compared with those in non-AKI patients, and AOPP levels were associated with AKI severity (Lentini et al. 2010).

The aim of the present study was to assess the prognostic performance of serum levels of AOPPs for the recovery of CSA-AKI after CABG.

## Material and methods

This observational study complied with the tenets of the Helsinki Declaration. It was approved by the Research Ethics Committee of Guangdong General Hospital (Guangzhou, China). Written informed consent was obtained from each participant.

### Study population

We retrospectively investigated all the subjects undergoing CABG between September 2007 and December 2008 from a prospective cohort in the Guangdong Cardiovascular Institute (which is one of the three major cardiovascular centers in China). In these patients, creatinine levels in serum were assessed every day for 7 days as a routine measurement for monitoring AKI. Exclusion criteria were: history of preoperative renal replacement therapy (RRT); estimated glomerular fltration rate (eGFR) <15 mL/min/1.73 m^2^; previous cardiac surgery; emergency or salvage CABG; potential pulmonary, endocardial or urinary infections (according to local protocols; by clinical manifestations; blood and urine examination; X-ray and cardiac ultrasonography) before hospital admission or during hospital stay; death of the patient; or loss of the patient to follow-up <3 months after surgery.

### Defnitions

Data obtained at the closest time point before surgery were defned as “baseline data”. The stratifcation of cardiac risk was evaluated by the European System for Cardiac Operative Risk Evaluation (EuroSCORE) with slight modifcations to the defnition of neurological disease, recent myocardial infarction and emergency surgery. A close approximation of the modifed edition had been validated (Zheng et al. 2009). Renal function was evaluated by the eGFR, which was calculated by the modifed diet and renal disease (MDRD) formula for Chinese populations (Ma et al. 2006). AKI was diagnosed and graded by the Acute Dialysis Quality Initiative (ADQI) consensus of RIFLE criteria based on serum creatinine levels (Bellomo et al. 2004):

- “Risk”: increase in creatinine of 1.5–2.0-fold from baseline.
- “Injury”: increase in creatinine of 2–3-fold from baseline.
- “Failure”: increase in serum creatinine of >3-fold from baseline.
- “Lose”: need for RRT for >4 weeks.
- “End-stage kidney disease (ESKD)”: need for dialysis for >3 months.

**Figure 1:**
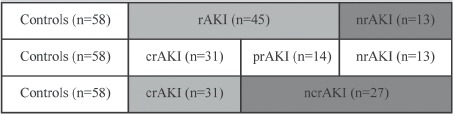
Renal outcomes according to diferent defnitions. rAKI, recovered AKI; crAKI, complete-recovered AKI; nrAKI, non-recovered AKI; ncrAKI, non-complete recovered AKI.

Recovered AKIs (rAKI) were included as completely recovered AKI (crAKI) and partially recovered AKI (prAKI) using RIFLE criteria at 3 months. crAKI was observed if patients returned to their baseline classifcation within the RIFLE criteria. prAKI was noted if there was a persistent change in RIFLE classifcation (R, I, or F). Non-recovered AKI (nrAKI) was observed if there was a persistent need for RRT (Bellomo et al. 2004). As a result, non-completely recovered AKI (ncrAKI) was composed of prAKI and nrAKI (Figure 1). nrAKI and ncrAKI were defned as adverse outcomes of AKI recovery.

### Laboratory measurements

Serial serum samples were obtained from patients with AKI at baseline, 2 days, 7 days, and 3 months, and from controls at baseline, 2 days, and 7 days, after surgery. Fresh serum samples were used for the measurement of serum creatinine, whereas samples for the measurement of AOPPs were stored at –80°C until use. Serum AOPP levels were determined as described previously (Witko-Sarsat et al. 1998; Chen et al. 2011). Briefy, 200 µL of serum was diluted 1:5 in phosphate-bufered saline (PBS) and mixed with 20 µL of acetic acid. In the standard wells for creation of the standard curve, 10 µL of potassium iodide (Sigma-Aldrich, St Louis, MO, USA) was added to 200 µL of chloramine-T solution (Sigma-Aldrich) followed by 20 µL of acetic acid. The absorbance of the reaction mixture at 340 nm was analyzed in a microplate reader (Thermo Multiskan MK3, Vantaa, Finland) within 3 min of adding acetic acid. AOPP levels were expressed as µmol/L of chloramine-T equivalents.

### Statistical analyses

Continuous variables are mean values ± SD or medians (25th and 75th percentile) and categorical data are percentages. Multiple groups were compared using the χ^2^ test for nominal variables. ANOVA (with Bonferroni correction) or the Kruskal-Wallis test was employed for numerical variables if appropriate. The AOPP level at baseline and after surgery was compared using the paired *t*-test. Spearman's correlation coefcient (*r*_s_) was used to assess the correlation between two variables. Univariate and multivariate logistic regression analyses were done to assess the efect of AOPPs on the adverse outcomes of renal recovery. Odds ratios (ORs) and their associated 95% confdence intervals (95% CIs) were estimated. To compare the discriminatory value of AOPP levels for renal recovery, a receiver-operating characteristic (ROC) curve was plotted and the areas under the ROC curve (ROC AUC) calculated. Statistical procedures were undertaken using SPSS software (version 13.0; SPSS, Chicago, IL, USA). Two-tailed tests were carried out for all comparisons. *p* < 0.05 was considered signifcant.

## Results

### Patient characteristics and outcomes

A total of 185 patients fulflled the inclusion criter ia (including 58 patients with AKI). From 127 subjects without AKI, 58 controls were selected randomly. Baseline characteristics of the controls and patients with AKI are shown in Table 1. Patients with AKI were older and had a higher urine albumin–creatinine ratio and higher EuroSCORE than controls. There were 22 in RIFLE R, 17 in RIFLE I, and 19 in RIFLE F in the 58 patients with AKI. Among them, 4 patients with RIFLE I and all the 17 patients with RIFLE F received RRT, and 13 patients had nrAKI and 27 patients had ncrAKI 3 months after surgery. These patients with AKI were further divided into two groups according to renal recovery (Tables 2 and 3). Patients with nrAKI were more likely to be older and to have a longer CPB time compared with patients with rAKI. Patients with nrAKI were older and had a higher EuroSCORE compared with those with ncrAKI.

**Table 1:** Demographic, clinical characteristics and blood chemistry data at baseline.

	Total (*n* = 116)	Controls (*n* = 58)	AKI (*n* = 58)	*p* value
Age (years)	63 ± 7	61 ± 6	64 ± 7	.004
Females (%)	45.7	40.0	55.2	.094
Estimated GFR (mL/min/1.73 m^2^)	86.3 (67.8, 93.2)	88.2 (79.0, 93.4)	84.4 (50.3, 93.6)	.228
Left ventricle ejection faction (%)	51.0 (37.3, 55.6)	51.2 (41.1, 55.9)	51.0 (33.1, 55.1)	.303
Cardiopulmonary bypass time (minutes)	118 ± 23	122 ± 24	115 ± 23	.079
Urine albumin–creatinine ratio (mg/g)	18.9 (11.1, 32.7)	14.7 (9.9, 25)	24.1 (12.2, 38.9)	.013
Diabetes (%)	24.1	17.2	31.0	.083
EuroSCORE score (point)	5 (3, 8)	4 (3, 6)	6 (4, 8)	.002

**Table 2:** Clinical features between diferent groups with recovered AKI and non-recovered AKI.

	nrAKI (*n* = 13)	rAKI (*n* = 45)	*p* value
Age (years)	68 ± 7	63 ± 7	.039
Female(%)	51.1	53.8	.862
Estimated GFR (mL/min/1.73 m^2^	78.3 (37.9, 92.3)	85.4 (56.6, 96.5)	.182
Serum creatine on day 7(µmol/L)	328.7 (124.3, 785.4)	295.2 (139.6, 687.5)	.232
Left ventricle ejection faction (%)	46.4 (28.9, 52.9)	51.2 (33.5, 55.6)	.292
Cardiopulmonary bypass time (minutes)	134 ± 17	119 ±24	.035
Albumin-creatinine ratio (mg/g)	17.5 (14.3, 30.7)	25.1 (11.5, 40.1)	.199
Diabetes (%)	46.2	26.7	.319
EuroSCORE score (points)	8.2 ± 4.6	6.2±3.4	.093

**Table 3:** Clinical features between groups with completely recovered AKI and non-completely recovered AKI.

	ncrAKI (*n* = 27)	crAKI (*n* = 31)	*p* value
Age (years)	65 ± 6	64 ± 8	.366
Female (%)	48.4	55.6	.586
estimated GFR (mL/min/1.73m^2^)	84.3 (44.7, 96.3)	84.4 (56.5, 92.1)	.919
Serum creatine on day 7 (µmol/L)	308.7 (174.3, 815.3)	275.1 (149.5, 787.5)	.132
Left ventricle ejection faction (%)	40.2 (28.9, 51.7)	53.4 (38.2, 55.9)	.011
Cardiopulmonary bypass time (minutes)	120 ± 22	124 ± 25	.621
Albumin-creatinine ratio (mg/g)	16.5 (10.4, 31.1)	25.1 (16.4, 41.1)	.114
Diabetes (%)	37.0	25.8	.356
EuroSCORE score (points)	8 (7, 9)	4 (3, 6)	<.001

### Kinetics of AOPPs

**Figure 2:**
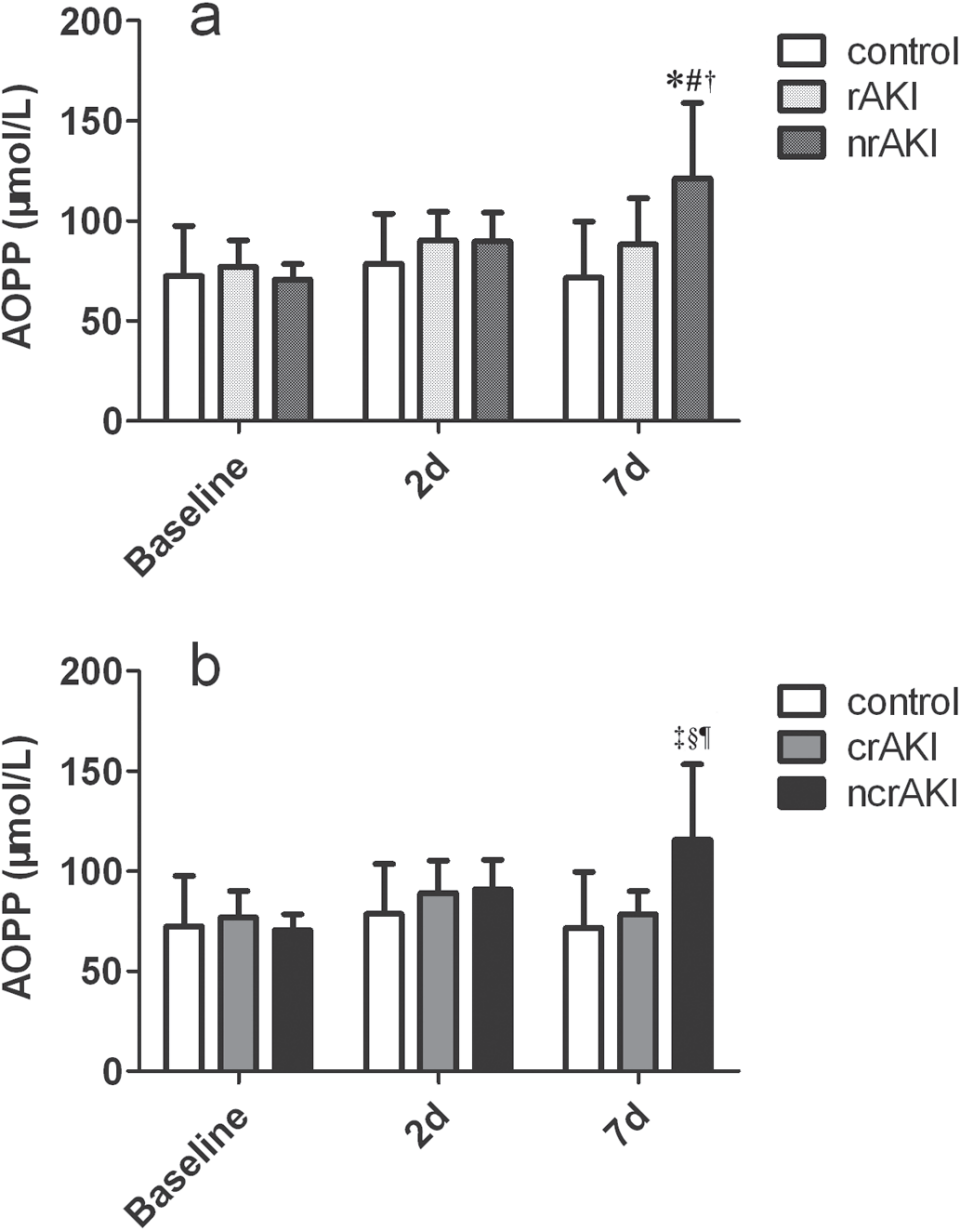
Kinetics of AOPP level in diferent groups. AOPP levels at baseline, days 2 and days 7 after surgery in controls, patients with recovered AKI and patients with non-recovered AKI (a) or in controls, patients with completely recovered AKI and patients with non-completely recovered AKI (b). rAKI, recovered AKI; crAKI, complete-recovered AKI; nrAKI, non-recovered AKI; ncrAKI, non-completely recovered AKI. *p < .05 AOPP level between patients with nrAKI and controls. #*p* < .05 AOPP level between patients with nrAKI and patients with rAKI. †*p* < .05 AOPP level between the baseline and at 7 days in the patients with nrAKI. ‡*p* < .05 AOPP level between patients with ncrAKI and controls. §*p* < .05 AOPP level between patients with ncrAKI and patients with crAKI. ¶*p* < .05 AOPP level between the baseline and at 7 days in the patients with ncrAKI.

AOPP level at 7 days increased by ≃50 µmol/L in the nrAKI group and increased by 40 µmol/L in the ncrAKI group after surgery. Patients with nrAKI had a higher AOPP level 7 days after surgery than patients with rAKI and controls (121.2 ± 37.9 *vs* 88.3 ± 22.9 and 71.7 ± 28.0), as well as patients with ncrAKI than patients with crAKI and controls (115.5 ± 32.4 *vs* 78.4 ± 11.6 and 71.7 ± 28.0) (Figure 2). Given that the levels of AOPPs at baseline and 2 days after surgery were comparable among groups, the results of the following two-time points are not shown. The AOPP level of all the patients with AKI and of controls 7 days after surgery was correlated to EuroSCORE values and CPB time, and had a mild inverse correlation with the baseline values of left ventricular ejection faction (Table 4).

**Table 4:** Relationship between AOPP level at 7 days and clinical indices.

	*r*_s_	*p* value
Age	0.108	.247
Left ventricle ejection faction	–0.227	.014
eGFR	–0.156	.096
Cardiopulmonary bypass time	0.159	.088
EuroSCORE	0.510	<.001

### AOPP levels as prognostic risk factors for adverse outcomes of AKI recovery

To evaluate the independent efect of AOPP level at 7 days on nrAKI or ncrAKI, AOPP level and variables with *p* < 0.10 in the univariate analysis (Tables 2 and 3) were candidates for the multivariable logistic analysis. An increased level of AOPPs of 10 µmol/L was related to an increased prevalence of nrAKI and ncrAKI of 50.9% and 188.3%, respectively (Table 5).

**Table 5:** AOPP as risk factors for adverse outcomes of AKI recovery.

	For non-recovered AKI				For non-completely recovered AKI			
		
	Univariable model		Multivariable model		Univariable model		Multivariable model	
				
	OR	95% CI	adjusted OR^a^	95% CI	OR	95% CI	adjusted OR^b^	95% CI
AOPP at 7 days	1.421	1.126–1.794	1.509	1.102–2.067	3.217	1.658–6.242	2.883	1437–5.787

a Adjusted by age, cardiopulmonary bypass time and EuroSCORE.

b Adjusted by left ventricle ejection faction and EuroSCORE.

### Prognostic performance of AOPP for adverse outcomes of AKI recovery

In the patients with AKI, the AOPP level at day 7 was a good biomarker for predicting nrAKI (nrAKI *vs* rAKI) and ncrAKI (ncrAKI *vs* crAKI) using ROC curves analyses. The prognostic strength of AOPP levels for adverse outcomes of AKI recovery is shown in Table 6.

**Table 6:** Predictive performances of serum AOPP at day 7 for adverse outcomes of AKI recovery.

	AUC	AUC 95% CI	Sensitivity	Specificity	Positive predictive value	Negative predictive value
AOPP for non-recovered AKI	0.791	0.644–0.937				
80			92.3	44.4	32.4	95.2
90			69.2	71.1	40.9	88.9
100			53.9	84.4	50.0	86.4
AOPP for non-completely recovered AKI	0.903	0.822–0.984				
80			92.6	61.3	67.6	90.5
90			74.1	93.6	90.9	80.6
100			48.2	96.8	92.9	68.2

## Discussion

Patients with poor recovery from AKI had a higher AOPP level, and an increased AOPP level was associated with nrAKI and ncrAKI. These fndings demonstrating that the AOPP level was a potential candidate for predicting adverse recovery from AKI. ROC curve analyses confrmed that the AOPP level had good prognostic value for predicting adverse outcomes after AKI.

Pre-existing proteinuria is associated with CSA-AKI (Huang et al. 2011). However, the urine albumin-creatinine ratio was not associated with renal recovery in the present study. Given that tubular injury, renal hemodynamics and intestinal infammation have key roles in renal recovery, albuminuria (a classical biomarker of glomerular injury) might not be a marker for recovery from AKI. No authors have demonstrated that the level of albuminuria is a risk factor for renal recovery. At the very least, the present study suggested that albuminuria was not a strong prognostic factor for renal recovery.

The lifespan of oxidized albumin (the main component of AOPPs) is shorter than that of native albumin (Iwao et al. 2006), but the kinetics of AOPP in CABG patients has not been investigated. We studied AOPP levels at 2 days and 7 days after CABG. The AOPP level at day 7 but not at day 2 had good prognostic value for renal recovery, but an optimized time point for measurement of the AOPP level needs to be investigated.

There were some limitations to our study. Results were from a relatively small number of patients in a single center. Tus, the results will need to be validated in a larger population. In addition, our study focused only on the AKI recovery after CABG. As a result, the serum level of AOPPs in controls undergoing CABG in the present study was higher than that of health controls in the same center (Chen et al. 2011; data not shown). We were not able to generalize our results to all cases of AKI. However, this homogenous study is possibly a better model for the prediction of adverse AKI recovery in such a population with a higher baseline level of AOPP. Finally, renal outcomes were followed up for only 3 months based on the suggestion of ADQI consensus. Late recovery of AKI after 3 months in patients requiring RRT is also recognized, but it is uncommon (Siddiqui et al. 2008). Therefore, the value of AOPP levels for the long-term recovery from AKI remains to be validated.

In conclusion, increased AOPP levels 7 days after CABG were associated with adverse outcomes of AKI recovery (nrAKI and ncrAKI) in CABG patients in a Chinese population. AOPP levels had good prognostic value for predicting adverse renal recovery.

## Declaration of interest

This work was supported by the National Natural Science Foundation of China (81170683) and the grant of Science and Technique Project of Guangdong Province, China (2010B031600157).
